# Relational developmental systems metatheory: a conceptual framework to understand and promote older adults’ involvement in sport

**DOI:** 10.1186/s11556-017-0182-6

**Published:** 2017-07-25

**Authors:** Amy M. Gayman, Jessica Fraser-Thomas, Joseph Baker

**Affiliations:** School of Kinesiology and Health Science, York University, 4700 Keele St, Toronto, ON M3J 1P3 Canada

**Keywords:** Relational developmental systems metatheory, Sport, Older adults, Individual factors, Contextual factors

## Abstract

Sport is viewed as a vehicle to develop or augment adaptive developmental processes, resources, and experiences across the lifespan. However, research has acknowledged that sport participation is associated with costs as well as benefits in older adulthood. To fully understand the influence of sport participation on psychosocial and developmental outcomes in older people, insight into the dynamic and complex relationship between the individual and his/her environment is needed. This review proposes Relational Developmental Systems (RDS) metatheory as a conceptual framework to understand the outcomes of sport participation later in life. Knowledge of the mechanisms, processes, and bi-directional exchanges related to individual and contextual relations emphasised in RDS frameworks may help researchers gain an understanding of the means by which sport participation shapes developmental outcomes evident within and among older athletes. Key assumptions of the metatheory are introduced and discussed in relation to the sport setting. Specific examples from the literature on older athletes are presented to illustrate the relationship between individual and contextual factors on developmental outcomes. Finally, considerations for future research on the topic are proposed using an RDS lens to move the field forward.

## Background

Developed countries are facing the cusp of an unprecedented demographic shift in population ageing as a consequence of increasing life expectancy, declining fertility rates, and ageing of the ‘Baby Boomer’ generation [1–3]. Statisticians have projected that the number of adults age 60 and older living in the world will increase from 901 million in 2015 to 2.1 billion by 2050 [3]. This trend has generated concern regarding healthcare resources, medical costs, and institutionalization of a rapidly growing cohort of the ‘oldest old’, many of whom will experience chronic disease and disability as they age [4, 5].

Consequently, physical activity throughout the lifespan is an area of considerable interest to researchers, health practitioners, and policy makers world-wide. Participation in physical activity is advocated as a modifiable lifestyle factor associated with improved cognitive and physiological functioning, reduced risk of chronic conditions, psychological health and well-being, as well as enhanced quality of life in older adults [6–8]. Such benefits pertain not only to those who maintain physical activity involvement into older adulthood but also those who adopt a physically active lifestyle later in life [9]. Given the positive influence of physical activity on healthy ageing, global and national guidelines recommend that older adults (over the age of 65) engage in at least 150 min of moderate levels of physical activity per week [6]. Yet, data continue to indicate that older adults are more inactive than younger age groups [10] even though they report ample time for leisure pursuits [11]. In Canada, for example, over half of adults aged 50–79 years report being physically inactive during leisure time [10].

Among active older adults, engagement in leisure-time physical activity can range from low to moderate levels of habitual, non-competitive activity (e.g., walking) to high levels of vigorous exercise necessary to train and compete in sport (e.g., Masters running) [12–14]. Masters athletes’ high level of sport involvement, in particular, has captured the attention of researchers since this group continues, resumes, or initiates sport participation at a point in the lifespan when most people are physically inactive [15, 16]. Their exceptional commitment to sport in the face of challenges, including age-related performance declines and negative societal stereotypes regarding the ‘appropriateness’ of intense physical activity in older adulthood, is especially noteworthy [17, 18].

Researchers have also recognized that older athletes may reap psychosocial benefits from their participation in sport that differ from those associated with other forms of physical activity involvement later in life. Baker and colleagues’ [16] discussion paper on sport participation and positive development in older adults highlighted the potential of sport to promote positive psychosocial outcomes unique to this movement context. The authors suggested that:

Masters sport is a unique context that provides opportunities and outcomes that extend beyond regular involvement in physical activity, such as walking or doing an exercise class. Masters sport allows older people to regularly compete against others within a similar age range in a variety of individual and team activities. Despite competition being inherent in Masters sport, the core values and social discourse underpinning this movement are participation for ‘fun, friendship, and fitness’, which is quite similar to how youth sport is framed (p. 4).

More specifically, Baker et al. [16] contended that sport participation yields similar psychosocial benefits regardless of age and thus, conceptual frameworks related to positive youth development (PYD) through sport may offer insight into sport participation and development in later life. Development in this context refers to transferable life skills and physical competencies that (ideally) foster thriving and contribution to the community [19]. Sport is viewed as a vehicle to develop and/or augment adaptive developmental processes, resources, and experiences in youth and older adulthood alike [16]. Results of a recent systematic review on psychosocial outcomes of older adults’ sport indicated that sport can foster feelings of excitement, influence perceptions of well-being and competence, facilitate the development of new relationships, provide opportunities to be a role model, enhance social support, encourage health-promoting behaviour, and improve perceptual-cognitive abilities [20]. Although findings supported the notion that sport can promote enjoyable and challenging experiences, positive social interactions, success, and perceptions of competence across the lifespan, some psychosocial outcomes were specific to older adulthood, including the formation of an ageing identity and coping with growing older [20].

Such findings reinforce the assertion that development is lifelong [21] and sport provides an opportunity to promote positive development in older people [16, 20]. However, potentially adverse outcomes of sport participation must be recognized to ensure sport involvement facilitates healthy ageing [20]. Sport has the potential to promote health and well-being across the lifespan but it is not a panacea. Coakley [22] cautioned against adopting an ‘evangelist’ view of sport, which assumes “that sport, unlike other activities, has a fundamentally positive and pure essence that transcends time and place so that positive changes befall individuals and groups that engage in or consume sport” (pp. 306–307). Gayman and colleagues [20] found evidence that sport participation in older adulthood can lead to feelings of frustration or fear when performance declines, experiences of negative comments regarding the appropriateness of sport participation, and contention within the family as a result of the commitment needed to train and compete in sport. Likewise, Winterbotham and du Preez [23] acknowledged that attitudes towards ageing in older athletes can be maladaptive. In comparison to older exercisers, athletes involved in sport later in life emphasized fear of loss, reproduction of ageist stereotypes, and the use of sport to fight the ageing process. Researchers have questioned the implications of ageing identities, athletic injury, and career termination in older athletic populations recognizing that sport can lead to unhealthy, maladaptive outcomes like social withdrawal, depression, difficulty coping, and feelings of guilt, shame or worthlessness [16, 23].

Certainly, future research should address the costs as well as benefits related to older adults’ sport participation, to understand the effects on development across the lifespan and to determine if these outcomes (adaptive or maladaptive) are related to engagement in physical activity generally or unique to involvement in sport more specifically. However, a myopic focus on identifying the outcomes of sport participation prevents researchers from gaining a holistic understanding of *how* sport influences developmental outcomes [24–26]. The ability of sport to foster positive developmental outcomes in older adulthood may be contingent upon a number of individual and contextual factors. Reflecting on youth sport involvement, Coakley [22] observed that: “By itself, the act of sport participation among young people leads to no regularly identifiable developmental outcomes. Rather, outcomes are related to and dependent upon combinations of multiple factors” (p. 309). Studies should assess the contextual factors and individual processes that contribute to the benefits and costs associated with the sport setting to better understand the means by which sport results in psychosocial outcomes that extend beyond those offered by other types of physical activity. Coalter [25] maintained that “we require a better understanding about what sports and sports’ *processes,* produce what *impacts*, for which *participants* and in what *circumstances*” (p. 20). Insight into the complexity of sport late in life will better inform the structure, promotion, delivery, and effectiveness of future programs and policies aimed at enhancing physical activity in older adulthood.

The objectives of this paper are to extend Baker and colleagues’ [16] discussion on the potential of sport as a distinct form of physical activity to influence developmental outcomes in older people and to offer a conceptual framework to better understand how the sport context coacts with characteristics of the individual to result in adaptive or maladaptive psychosocial outcomes. Specifically, the key assumptions of Relational Developmental Systems (RDS) metatheory, which has been used extensively by developmental psychologists to study the influence of individual and contextual factors on human development, will be applied directly to the sport setting. Research on older adults’ involvement in sport will be reviewed to illustrate the potential of RDS to serve as a conceptual framework that can be used to understand psychosocial outcomes of sport participation in older adulthood. Finally, considerations for future research will be addressed within an RDS framework in an effort to move the field forward.

## RDS and positive development through youth sport

RDS has been upheld as a metatheoretical framework that can integrate and drive research on PYD across movement contexts, including sport [24, 27, 28]. The fundamental assumption underlying RDS is that change can occur throughout the lifespan [29]. Central to understanding developmental changes over time is the notion of relative plasticity. From birth until death, individuals change in both quantitative and qualitative ways [30]. Although some processes and variables remain the same across an individual’s life, and cultural, sociohistorical, and physical constraints limit the extent of change over the lifespan, developmental trajectories are not fixed, nor are developmental systems ever completely constrained [31, 32]. Plasticity in the developmental system permits change in every stage of life [30, 31, 33] and is viewed as a “fundamental strength of human development” (p. 4) [33]. Accordingly, lifespan developmental theorists are primarily interested in identifying, explaining, and enhancing discontinuity and continuity of change over time [30, 31, 33].

Developmental changes seen between and within individuals at various points in the life course are believed to be regulated by the dynamic, reciprocal exchanges between individual and contextual factors. The pace, direction, and outcomes of development are influenced by this relationship between the person and his/her environment. Changes in development may be beneficial or detrimental depending on the bidirectional relationship between individual and contextual factors and differences in developmental trajectories (across individuals and time) hinge upon the exchanges between individual and contextual variables that ultimately shape one’s development [29, 30, 33]. RDS models, therefore, focus on understanding the complex and varied relations between characteristics of the individual and of his/her surrounding environment [29, 30]. It is assumed that the complexity of developmental systems and plastic nature of individual and contextual relations present an opportunity to nurture adaptive developmental regulations, which benefit both person and environment [30, 33].

This optimistic view of development across the lifespan is the cornerstone of PYD models adopted to explain and promote positive development in adolescence:

a key assumption of relational developmental systems theories – and, as we will note, of the use of these theories to understand both adolescent development in general and to frame the PYD conception of developmental processes more specifically – is that the developmental system is sufficiently diverse and complex such that some means may be found (by researchers and/or practitioners) to couple individual and context in manners that enhance the probability of change for the better, of promoting more positive features of human development (p. 4) [33].

To manipulate and encourage the positive development of youth through organized activities (including sport), an in-depth understanding of the nuances of person-context relations that influence developmental outcomes is essential. RDS underpinnings of PYD models emphasize the need to identify the contextual factors and personal strengths that lead to certain outcomes in youth [34] and how such outcomes then cultivate other adaptive developmental regulations [33].

In the sport setting, Agans and colleagues have highlighted the need to study variation in athletic experiences that occur as a result of differences in the characteristics of participants (e.g., behaviour, attitudes, skills), the activity context (e.g., type of sport, coaching style, team culture, motivational climate) and the interplay of these factors to understand PYD [24, 35]. Such experiences within the relational developmental system are inextricably tied to the concepts of personal agency and embodiment [24, 27]. The individual is recognized as an active agent with the capacity to influence the context and in turn, his/her own development through adaptation, self-organization, and/or self-regulation [31, 32, 36]. Young people are not only influenced by the environments in which they are embedded, but also play a pivotal role in their own development, shaping the environment in ways that are mutually beneficial for the individual and the context by optimizing resources to align with their interests, desires, and needs [37–41].

Given the individual’s active engagement with the world, his/her lived experience of individual-contextual relations underlying development is important to recognize. Overton asserted that: “all acts are embodied acts, and consequently the general case is that it is not simply acts, but rather it is *embodied action* that *constitutes the fundamental process for all developmental change*” (p. 50) [42]. The concept of embodiment emphasizes the body not only as an observable, physical object but also as a psychological experience in which the individual’s relations with his/her surroundings in his/her particular body influences thoughts, feelings, and behaviour [36, 42, 43]. A recent examination of female swimmers’ lived experiences of body practices, for instance, exemplifies the concepts of personal agency and embodiment in sport. Swimmers described self-regulatory efforts (e.g., dieting, increased training) to alter their sexually maturing bodies in an attempt to achieve the boyish physique associated with success and acceptance in the culture of high performance swimming. Their struggle with a particular type of body (i.e., feminine) in a sporting environment, ultimately resulted in feelings of shame, emotional insecurity, self-doubt, physical tiredness, and confusion regarding physical development [44].

The dynamic and complex relationship between the active, embodied individual and context is fundamental to understanding developmental outcomes of sport participation [24]. Indeed, researchers have acknowledged that the benefits of sport participation may be dependent upon personal and contextual factors such as the organization and delivery of programs, cultivation of positive relationships, development of internal/external assets, knowledge and competencies, promotion of meaningful personal experiences, individual perceptions of the sport context, and integration of sport experiences in other spheres of life [22, 45–48].

Although the benefits of sport participation are contingent upon how sport is experienced and delivered [22, 48, 49], PYD research and programming have been criticised for failing to identify the mechanisms, processes, and relations that result in adaptive developmental outcomes [25, 26, 40, 50–52]. Sport is hailed as an ideal context to promote PYD; however, sport participation in and of itself does not necessarily lead to positive developmental outcomes [22]. Additional information regarding the mutually influential relationship between individual and contextual factors is needed to fully understand the manner by which sport results in PYD [24]. Nonetheless, researchers typically adopt a variable-centred perspective studying sport as a predictor or moderator variable or focus exclusively on outcomes related to PYD programs involving sport [24, 26]. This essentially creates a ‘black box’ that provides little understanding of the characteristics, experiences, processes, and mechanisms that account for why PYD programs related to sport are associated with particular outcomes and does not explain how such outcomes may be unique to specific sport contexts (e.g., individual vs. team, recreational vs. competitive) [24, 26, 41, 49, 53]. Greater emphasis on the lived experience of the individual as an active contributor to developmental change, situated within a mutually influential and adaptable context is needed to gain a holistic understanding of developmental outcomes in youth sport participation and should be considered by those interested in applying PYD models to explain the benefits (or costs) experienced as a result of sport involvement in older adulthood.

## RDS and developmental outcomes of sport for older people

In an editorial devoted to studying older adults’ physical activity, Whaley [54] challenged researchers to adopt a developmental perspective to better “understand intraindividual change (change within older adults over time) and interindividual differences (differences among older adults)” (p. 301). RDS suggests differences observed within and between individuals over time are due to the plastic nature of development, which is related to variation in individual and contextual relations. As such, policies and programs may be created to advance positive, healthy development by capitalizing and aligning strengths of individuals (e.g., self-regulation skills, hope, engagement) and the contexts of which they are a part (e.g., home, school, community) [30, 33, 55, 56]. Knowledge of the mechanisms, processes, and interplay of individual and contextual relations emphasised in RSD frameworks may help researchers gain insight into the means by which sport shapes differences in developmental outcomes evident within and among active older people. Researchers must ascertain *what combinations* of individual attributes and contextual conditions *at which points* in older adulthood induce variations in developmental outcomes [29].

A comprehensive understanding of the individual and contextual factors associated with developmental change (either adaptive or maladaptive) as a result of sport in older adulthood will inform efforts to promote favourable developmental outcomes and avoid potentially adverse effects of older adults’ sport involvement. The following section presents examples of existing research on individual and contextual factors related to older adults’ involvement in sport to illustrate the potential of RDS to serve as a conceptual framework that can be used to understand and promote adaptive development through sport participation later in life.

### Individual factors related to outcomes of older adults’ sport involvement

A main principle of RDS is that individuals, as active agents, have a significant impact on their own development and can affect the environment in which they are embedded. Individual factors including biological, demographic, personality, cognitive, motivational, emotional, and behavioural characteristics in conjunction with context-specific variables explain variation or consistency in developmental trajectories over time [29–32].

Cardenas, Henderson, and Wilson [57] suggested demographic characteristics may affect older adults’ perceptions of the benefits of sport. Perceived health and physical strength, self-esteem, and likelihood of meeting people were rated more highly among Senior Games participants who lived alone, possessed high school or lower levels of education, and reported an income of $19,000 or less. Age and sex have also been associated with motivation as well as fears surrounding ageing and performance. In contrast to younger cohorts, older athletes contend with age-related declines typical of old age and report participating in sport to delay or prevent the loss of physical abilities, mental functioning, and independence [58–61]. Such concerns, however, may differ between men and women. Eman [61] found women were less likely to fear ageing because they focused on personal capabilities and evaluated performance in terms of stamina, discipline, and experience. Older male athletes emphasized the importance of sport to the maintenance of the “continuous self in the process of aging” (p. 470) and based judgements of ability on performance records and comparisons to others. Previous work on achievement motivation in middle-aged and older Masters athletes has indicated that high task orientation (indicative of a focus on mastery and personal competence) was associated with adaptive motivational patterns [62, 63]. Perhaps this explains why research has reported that older female athletes experienced more enjoyment in sport [64] and perceived greater benefits of their participation than their male counterparts [57].

Outcomes of sport participation later in life may also be influenced by personality attributes such as competitiveness or passion towards sport and exercise. It has been suggested that competitiveness may play a mediating role in encouraging adaptation (e.g., selective optimization) to physical and environmental limitations on performance typical of ageing and in turn, continued successful involvement in sport [65]. The experience of adaptive sport outcomes, however, may be dependent upon the level and type of attribute expressed by the individual. Young et al. [66] studied the impact of passion among a sample of World Masters Athletics Championship participants and found the effects of sport participation varied according to the type of passion internalized by the individual. In contrast to harmonious forms of passion, obsessive passion was related to negative emotions (i.e., disappointment, restlessness, irritability, guilt and anger), high levels of self-pressure, amotivation, intent to reduce involvement in sport, and conflict between devotion to sport and other life commitments.

In addition, the importance of recognizing the impact of previous athletic experience and skill/performance level has been advocated in the literature. Baker, Côté, and Deakin [67] emphasized the need for research to clarify the influence of different training and developmental profiles between expert and non-expert older athletes. The authors acknowledged that differences in psychological and physiological characteristics are likely to exist between elite and non-elite athletes (e.g., competitive vs. recreational level) even among ageing populations and that such differences may influence health outcomes related to participation in sport [67]. Athletic experience and level of sport participation have been associated with sport motivation. Athletes who reported long-term involvement in competitive sport (i.e., youth competitive sport experience) were more likely to stress the importance of achievement than skill development in comparison to novice athletes with less competitive experience [64]. However, the emphasis on achievement in competitive sport may lead to varying outcomes depending on the level of competitive sport involvement. De Pero and colleagues [68] found higher levels of self-determined motivation in elite level athletes (i.e., national or international competitors) over the age of 65. In comparison to those competing at non-elite levels (i.e., local/regional competition) elite athletes were more intrinsically motivated [68]. These findings are interesting given that high levels of competition in youth sport are associated with lower levels of intrinsic motivation and amotivation. The authors acknowledged that motivation in sport is specific to one’s stage of life and emphasized the need to understand varying levels of motivation among older athletes to encourage sport commitment and lifelong participation in sport [68].

Athletic experience may also influence identity development. Participation in sport late in life may represent a pattern of lifelong involvement, a return to previous sport activity, or the beginning of a new leisure pursuit. Researchers should be cognizant of an individual’s sporting history to gain insight into potential disparities in sport experiences and outcomes of participation including the effect on one’s sense of identity. Lifelong athletes and those who have resumed sport participation maintain (or reclaim) an athletic identity [69–72] and have admitted a sense of empowerment, self-competence, enjoyment [69] and satisfaction that they “can still do it”! (p. 20) [70]. However, some older athletes may experience high levels of self-pressure and feel obligated to maintain their involvement in sport because they fear relinquishing their identity as an athlete [66, 73]. Newcomers to sport, on the other hand, construct a new or alternative identity that positions them as a sportsperson, teammate, or physically active individual [70, 74]. The formation of an adaptive identity as an athlete has been viewed as positive or liberating and is associated with personal competence, achievement, and status [69]. Of course, more research on older athletes who drop out of sport is needed to determine the effect of declining performance results typical of ageing on athletic identity. Baker et al. [16] speculated that some athletes may find it difficult to cope with an ageing identity. This underscores the importance of studying individual factors and differences in lived experiences to fully understand the benefits and/or costs associated with sport for older people.

### Contextual factors influencing outcomes of older adults’ sport involvement

Developmental outcomes, according to RDS frameworks, also vary in relation to a person’s environment. It is the dynamic, reciprocal relations between a combination of contextual and individual variables that ultimately results in developmental change within and across people over time. In particular, setting (e.g., family, peer group, neighbourhood, community, workplace), cultural, institutional and social-historical variables may promote or detract from adaptive personal development [29, 30, 33]. This means that the environment created by coaches, peers, and other significant members of the sport network is critical to the formation of favourable (or deleterious) outcomes in athletes [45, 48].

The social context of sport for older people is particularly influential. Sport offers an opportunity to not only broaden social networks but also reaffirm previously established relationships. A number of studies have recognized that sport participation is valued by participants because it increases social engagement, interaction, and affiliation with others [59, 70, 71, 75, 76]. Positive social contexts characterized by supportive social relationships in sport are associated with more positive mood, greater functional commitment to sport, and an enhanced sense of community [76–79]. In contrast, social constraints including family members’ disapproval of athletes’ commitment to sport resulted in experiences of tension at home, and perceptions of pressure from spouses [80], children, coaches, and/or teammates was associated with feelings of obligation to maintain sport involvement [73, 79, 81].

Older adults have also recognized the importance of competition within the context of sport. Competitive sport permits older people to remain involved in sport regardless of their age or performance level and participation is characterized by a narrative of acceptance, adaptation, and resolve [59]. Within this context, decrements in performance as a result of ageing are embraced [59] and older people are able to adapt to limitations typical of an ageing body by altering their play, changing their level of involvement, maintaining their physical fitness, or becoming involved in a different sport [59, 65, 82]. Further, continued participation in competition later in life may help older people redefine the ageing process by challenging traditional societal stereotypes associated with growing older [16, 58, 60, 61, 69]. While resistance to negative stereotypes of ageing has been linked to empowerment and developing alternative identities [69], some older athletes have expressed feelings of embarrassment, guilt, or avoidance of the notion that they are competitive [12, 60].

Others have recognized that sport later in life satisfies competitive needs since it offers opportunities to overcome challenges and experience personal achievement; thereby, fulfilling a need to compete [69, 83]. Ongoing pursuit and attainment of personal goals motivates older people to keep fit and healthy, encourages personal growth, and promotes greater effort [70, 76, 82, 83]. The competitive element of sport is valued by older athletes since it offers objective performance feedback in regards to personal differences in ability over time and in relation to others within the same age cohort [12, 70]. Winning medals, breaking records, recognition, and status gained from success in competition has been recognized as central to older athletes’ sport enjoyment, self-esteem, and prolonged engagement in sport [12, 60, 71]. Competition is viewed as challenging and the demands of sport encourage older people to exert themselves to the utmost of their abilities [70, 83]. As a result, self-confidence is gained when older adults are able to push their bodies beyond perceived limits [72]. In developing skills and realizing personal achievements in sport, intrinsic motivation, enjoyment, sport commitment, as well as, a sense of mastery, competence, satisfaction, empowerment, and athletic identity are enhanced [62, 69, 72, 77]. Such outcomes illustrate the promise of sport in terms of positive development in older adulthood. Yet, it is important to note that participation alone does not foster positive developmental outcomes [22]. Positive development is not inevitable but depends on the structure, delivery, and experience of sport for older people and how contextual factors coact with personal characteristics of the individual.

## Future directions and conclusion

Baker et al. [16] suggested that sport can promote positive or negative outcomes in older adults similar to those documented in research on youth development through sport. The present review supports and extends this argument by using RDS as a conceptual framework to understand how the sport context coacts with individual characteristics to influence developmental outcomes in older adulthood. The reviewed literature on sport for older adults has indicated that individual factors such as living arrangement, level of education, income, age, sex, competitiveness, passion, athletic experience, and skill/performance level may influence perceived health and physical strength, self-esteem, enjoyment, motivation, sport commitment, identity, and perceptions of competence. In addition, the social context and element of competition inherent to sport can contribute to developmental outcomes experienced by older athletes related to athletic identity, enjoyment, sport commitment, motivation, confidence, personal growth, and empowerment. In the section below we highlight some key areas of future research.

## The need for continued examination of the interrelationships between individual and environment

Although studies reviewed were not explicitly designed to examine the influence of individual or contextual factors on psychosocial outcomes in older sport participants, findings affirm the need to investigate the interrelationship between the active, embodied individual and the adaptable environment to understand beneficial (or detrimental) psychosocial outcomes in older athletes. Youth sport research has focused exclusively on participation as a predictor or moderator of other variables [24]. We argue that the literature on sport for older adults has adopted a similar approach to research. To move the field forward and gain a holistic understanding of the mutually influential relationship between person and environment, simultaneous investigation of multiple variables is required to better understand the dynamic and complex process of development [29].

## The need to expand sample diversity

Researchers are also urged to recruit participants of varying ages, demographic characteristics, cultural backgrounds, and sport involvement to understand development over time and across individuals. Research on sport in older adulthood has been criticized for focusing on highly educated, middle-class, Caucasian Masters athletes [70, 84]. Variables such as level of activity investment (e.g., recreational versus competitive), years of involvement, and time of sport adoption (e.g., continued, resumed, newly initiated) should be measured to account for differences in psychosocial outcomes later in life [14, 20, 66]. The influence of age on psychosocial outcomes of sport participation should be considered as well [20]. Many studies on older adults’ sport participation including those reviewed, have solicited a wide range of ages (from those in their early 50s to those over 65 years of age) [20], which does not address the diversity of lived experiences across and within various generations of older adults that may contribute to differences in development [54, 85, 86]. Increased attention to the influence of nationality is also warranted. Although data from the 2013 World Masters Games indicated that Masters sport is dominated by Western and European countries, competitors represented 105 countries [87]. As Masters sport programs continue to expand globally, greater diversity in the populations studied is recommended. The embeddedness of the individual within the larger social and cultural context may affect his/her lived experiences and future individual-contextual relations which account for healthy, positive development [27].

## The need for superior study designs

In addition, future studies in the area should consider longitudinal designs. Lifespan development assumes early events and processes are interconnected to developmental outcomes apparent in later stages of life [32]. In this respect, data on early adaptive (or maladaptive) experiences of older adults who continue, resume, or dropout of sport will provide insight into developmental outcomes later in life. Further, tracking athletes as they age will allow researchers to identify changes in developmental trajectories over time as well as consistency in developmental profiles. RDS frameworks recognize that lack of change is not necessarily indicative of a lack of plasticity in development but rather the result of individual efforts to maintain consistency in response to contextual constraints or demands [31].

Assessment of the dynamic and complex nature of individual-contextual relations, however, can be challenging for those conducting research on development that is informed by RDS metatheory. To study intraindividual change and interindividual variation in intraindividual change, researchers not only need to examine differences among and between individuals over multiple points in time, but also design and implement studies that involve a multi-level, multivariate, and multi-method approach [29]. While quantitative, experimental designs may provide valuable information on averages, they mask the nuances of individual-contextual relations. Mixed method, idiographic data analyses should also be implemented to gain insight into the uniqueness of the individual, diversity of lived experiences, and differences in developmental pathways. Such analyses may include dynamic factor analysis, idiographic filter, or narrative inquiry [29, 88].

## Greater attention to understanding athletes’ adaptations

Additional work on older athletes’ efforts to regulate and/or adapt to the sport environment is needed. RDS acknowledges that the individual is actively engaged in the process of development and has a significant impact on the environment through personal agency [31, 32]. A case study of a 68 year old competitive tennis player reported that the participant was able to maintain success in sport and continue to compete at a high level by implementing adaptive strategies and seeking out resources to compensate for physical and environmental limitations (e.g., activity choice, selection of playing partners, use of strongest skills, optimal scheduling, use of superior equipment, maintenance of physical fitness) [65]. However, we know little of older athletes’ effort or ability to shape the context of sport beyond actions to maximize their performance in the face of age-related physical decline. Nor have researchers thoroughly addressed the impact of older athletes outside of sport. The PYD literature argues that the experiences, resources, and skills young people possess or gain within contexts like sport leads to civic engagement and contribution to society [89, 90]. Masters sport researchers have debated whether older athletes serve as role models that inspire healthy, active living among peer groups [16] but specific instances of older athletes’ contribution to the larger community remain understudied.

## The need for multidisciplinary research teams

Lerner, Fisher, and Weinberg [91] called for descriptive and explanatory research conducted in context to gain insight into the processes involved in human development. To achieve this end, multi-disciplinary research teams are recommended [29, 88, 91, 92]. RDS frameworks maintain that the developmental system is comprised of many levels of integration including the biophysical, psychological, sociocultural and historical [30, 32, 89, 92]. It is the coaction of complex, interdependent processes from genes to culture that account for developmental change across the lifespan. The individual and context are mutually influential and as such, an integrated approach to research is encouraged to gain a holistic understanding of the relational processes underlying developmental outcomes [42]. Emerging work in epigenetics, for instance, has found that perceptions and experiences of social-environmental conditions (e.g., social isolation, subordination, rejection) can affect gene transcription and expression that leads to lasting behaviour changes and susceptibility to disease (e.g., cardiovascular disease, cancer, neurogenerative diseases, infectious diseases) [93]. Thus, interdisciplinary collaboration may provide a more comprehensive understanding of development through sport in older adulthood.

## Conclusions

In conclusion, substantial effort is required to build a body of knowledge that explains how sport leads to developmental outcomes later in life. Well designed, inter-disciplinary studies that uphold the assumptions of RDS frameworks may enlighten our understanding of this phenomenon. Such information is imperative to structure and deliver effective sport programming in older adulthood and to the development of policies and public health messages to promote physical activity and more specifically, sport later in life.

## Abbreviations

PYD: Positive youth development
RDS: Relational developmental systems
